# Nano-bioremediation of textile industry wastewater using immobilized CuO-NPs myco-synthesized by a novel Cu-resistant *Fusarium oxysporum* OSF18

**DOI:** 10.1007/s11356-022-23360-7

**Published:** 2022-10-03

**Authors:** Osama M. Darwesh, Hao Li, Ibrahim A. Matter

**Affiliations:** 1 Agricultural Microbiology Department, National Research Centre, 33 EL-Buhouth St., Dokki, Cairo, 12622 Egypt; 2 School of Chemical Engineering and Technology, Hebei University of Technology, Tianjin, China

**Keywords:** Myco-synthesized CuO-NPs, Industrial wastewater, Antibacterial activity, Textile dyes, Nano-bioremediation, *Fusarium oxysporum* OSF18

## Abstract

Currently, bionanotechnologies are attracting great interest due to their promising results and potential benefits on many aspects of life. In this study, the objectives was to biosynthesis CuO-NPs using cell-free extract(s) of copper-resistant fungi and use them in bioremediation of textile industry wastewater. Out of 18 copper-resistant fungal isolates, the novel fungus strain *Fusarium oxysporum* OSF18 was selected for this purpose. This strain showed a high efficiency in extracellular reducing copper ions to their nano-form. The myco-synthesized CuO-NPs were characterized using UV-Vis spectroscopy, HRTEM, FTIR, and XRD and were found to be spherical nanocrystals with the size range of 21–47 nm. The bio-synthesized CuO-NPs showed promising antimicrobial activity as well as high efficiency in removing heavy metals and textile dye from industrial wastewater. The myco-synthesized CuO-NPs immobilized in alginate beads exhibited superior microbial disinfection (99.995%), heavy metals removal (93, 55, and 30 % for Pb, Cr, and Ni, respectively), and dye decolorization (90%). Such results represent a promising step to produce an eco-friendly, cost-effective, and easy-to handle tool for the bioremediation of textile industry wastewater.

**Supplementary Information:**

The online version contains supplementary material available at 10.1007/s11356-022-23360-7.

## Introduction

Environmental pollution is currently increased rapidly as a result of urbanization, industrial revolution, and population growth. The textile and dyeing industries are among the industries that produce large quantities of wastewater (containing synthetic dyes and heavy metals). Several serious environmental problems occur when this polluted water is discharged into water bodies without adequate or appropriate treatment. Textile wastewater could be treated via different chemical, physical, and biological approaches or a combination thereof (Darwesh et al. 2019a; Chung et al. 2022). Nanomaterials (with one or more dimension ranged between 1 and 100 nm) have extraordinary properties due to their high specific surface area and distinctive electrochemical characteristics. Such properties increase the importance of nanoparticles and nanocomposites in various technological fields including biotechnology and environmental applications. Environmentally, these small-sized materials could play a critical role in element adsorption, catalytic decomposition of organic compounds, and microbial disinfection. Thus, it can be applied to treat various urban, industrial, agricultural, and industrial wastewaters (including textile wastewater) (Anjum et al. 2019; Darwesh et al. 2019b; Parvin et al. 2019).

Copper nanoparticles (Cu-NPs) have many potential applications in various fields such as electronics, optics, medicine, conductive films, lubricants manufacturing, nano-fluids, and antimicrobial agents (Powara et al. 2019). In addition to previous applications, Cu-NPs were applied for environment remediation and protection from various hazardous contaminants like textile dyes, phenolic compounds, heavy metals, and some pesticides (Ostaszewska et al. 2016). The Cu-NP preference is compared to silver that had ease of mixing with polymers, chemical, and physical stability and lower cost than silver (Vimbela et al. 2017). Many explanations were written about understanding the mechanisms of nanomaterials’ antibacterial activity like contacting with microbial cell wall, binding with DNA, or attached with microbial proteins (Slavin et al. 2017). Moreover, the charges of metal-based nanoparticles (MBNs) create strong bond with heavy metals as well as dye structure, resulting in component precipitation (Stensberg et al. 2011).

Several methods are applied for synthesis of MBNs like chemical, biological, and physical procedures. The produced nanomaterials may differ from method to other depending on various parameters such as reducing materials or system, capping agents, stabilizing substances, and environmental conditions (Hussein et al. 2019; Khan et al. 2019; Velsankar et al. 2020d). Chemical methods may produce toxic materials as undesirable substrates and may be done under uncontrolled reactions (Yu et al. 2009). Also, physical methods are highly costed and need especial instruments, in addition to unchanged properties upon bulk materials (Dhand et al. 2015). However, biological procedures are advantaged as safe, environmental-friendly, controlled, produce high-active nanomaterials, and have capping and stabilizing agents (Sultan et al. 2016; Velsankar et al. 2021). In this regard, bacteria, actinomycetes, microalgae, and fungi have been reported for the green biosynthesis of different nanoparticle (Darwesh et al. 2019b). The dynamic goods of the copper led researchers to convert it to nano-forms for explication of its antimicrobial/antioxidant properties. The biosynthesis of antimicrobial CuNPs by *Streptomyces* spp. was mentioned by Hassan et al. (2018). Also, anticancer activity was successfully monitored for CuO-NPs and CuNPs produced by *Trichoderma* species (Saravanakumar et al. 2019).

Fungi, in particular, are known for their superior ability to produce many bioactive compounds, and their efficiency in converting metal ions into their nanoscale form (Fouda et al. 2021; Ahmed et al. 2022). Therefore, in this study, a number of fungi resistant to copper ions were selectively isolated from soil and then the most effective fungal strain in the biosynthesis of CuO-NPs was selected. Furthermore, the green synthesized CuO-NPs were characterized, immobilized in alginate beads, and investigated as a disinfectant, heavy metal adsorbent, and decolorizing agent for textile industry wastewater (synthetic and real).

## Materials and methods

### Isolation and screening of copper-resistant fungi

All isolation sources (soil and textile industry wastewater) for copper-resistant fungi were collected from Giza governorate, Egypt. The samples were seven samples of soil (three from copper foundries and four from neighboring agricultural farms) and five samples of wastewater (from textile factories in 6th of October City). The samples were collected and transferred to the lab under aseptic conditions and stored in refrigerator at 4 °C until used [18]. Isolation of copper-resistant fungi was set up using consecutive enrichment culturing technique. Briefly, 10 g/mL of soil/wastewater sample was (in triples) enriched in 250-mL conical flasks containing 100-mL potato dextrose broth medium (PDB) amended with 1 g/L CuSO_4_ (as enrichment medium). Flasks were incubated under orbital shaking conditions (100 rpm) at room temperature for 10 days. The enrichment cultures were successively repeated three times by transferring 10 mL of the previous culture to fresh enrichment medium and incubated under the same conditions (Hasanin et al. 2019). One-milliliter aliquot from each flask was then transferred to a 1%-saline water tube for progressive dilution to 10^−5^. Finally, 100-μL aliquot of suspension was coated on potato dextrose agar (PDA) plates supplemented with ampicillin (50 μg/mL) as anti-bacterial and 1000 mg L^−1^ CuSO_4_·5H_2_O and incubated at 28 °C for 7 days (Darwesh et al. 2020). Representative fungal colonies with different morphology on agar plates were isolated, purified, and maintained on the same isolation medium for further investigations.

Purified fungal isolates were screened on the basis of their tolerance to Cu^2+^. A disk of mycelium was inoculated aseptically on PDA plates supplemented with 1000 ppm CuSO_4_. The diameters of the emerging colonies (mm) versus the control group (medium without Cu^2+^) were measured after incubation at 28 °C for 7 days. Furthermore, the ability of fungal isolates to grow in liquid medium (PDB) supplemented with 1000 ppm CuSO_4_ was screened. Duplicates of 250-mL conical flasks containing 100 mL PDB-copper medium were inoculated with a disk of fungal mycelium and incubated under orbital shaking conditions (100 rpm) at room temperature for a week. The growth of fungal isolates was estimated by measuring the final dry biomass. Fungal isolates that showed good resistance and growth in the presence of copper were selected for subsequent experiments.

### Screening fungi for extracellular biosynthesis of copper NPs

The ability of copper-resistant fungal isolates to reduce and convert copper ions into an extracellular nanoform was screened. Into 250-mL conical flasks, 100 mL of PDB medium (pH 5.5) was inoculated with 1 mL of fungal spore suspension (10^6^ spore mL^−1^) and incubated at 100 rpm and 28 °C for 3 days. At the end of incubation period, the cultural filtrates were applied as biological reducing agent to transform Cu^2+^ into its nanostructures. The cultural filtrates have been mixed with an equal volume of 0.5% CuSO_4_ solution and incubated in dark for 3 days on a rotary shaker at 100 rpm at 28 °C. The biosynthesis of Cu-NPs was checked (daily) by measuring the absorbance values of the aforementioned mixtures at 570 nm using a UV spectrophotometer (Jenway UV/Visible 2605 spectrophotometer, England) (Khodaie and Ghasemi 2018). Based on the efficiency of CuO-NP formation and tolerance for copper ions (from previous experiment), the most promising fungal isolate was selected for identification and further investigations.

### Identification of selected fungal isolate for Cu-NP biosynthesis

Identification of the selected fungal isolate up to the genus level was carried out using morphological microscopic characteristics (Olympus cx41, Japan). Meanwhile, the molecular identification of the fungal species was carried out using sequencing of the ITS region. In brief, the total genomic DNA was extracted using CTAB protocol (Eida et al. 2018) for fungal mycelium harvested after cultivation in PDB medium for 3 days. Extracted DNA was amplified using polymerase chain reaction (PCR) by ITS1 (5′-TCCGTAGGTGAACCTGCGG-3′) and ITS4 (5′-TCCTCCGCTTATTGATATGC-3′) primers. The identification was achieved by comparing the contiguous DNA sequence with data from the reference and type strains available in public GenBank databases using the BLAST program (National Centre for Biotechnology Information) (http://www.ncbi.nlm.nih.Gov/BLAST). The obtained sequences were aligned using Jukes Cantor Model (Darwesh et al. 2019c). Finally, the sequences were deposited in GenBank and the accession number was obtained.

### Production and characterization of myco-synthesized Cu-NPs for environmental applications

The selected fungus (isolate F18) was cultivated for 3 days in a 3-L flask containing 2 L of PDB medium aerated by bubbling with sterile air. The cell-free culture filtrate was mixed with a similar volume of 0.5% CuSO_4_ solution and stirred for 24 h to allow the formation of Cu-NPs. The formed Cu-NPs were allowed to precipitate, pooled, and washed once with distilled water and twice with absolute ethanol and then dried at 50 °C. The dried and grounded Cu-NPs were subjected to further characterization before being used in antimicrobial and textile wastewater treatment experiments.

Cu-NPs biosynthesized using the fungus F18 were characterized by high-resolution transmission electron microscopy (HRTEM) (JEOL 2100 Japan) to define the size and shape of the produced nanostructures. Fourier transform infrared spectroscopy (FTIR) analysis was performed to determine the possible biomolecules guild for capping, reduction, and efficient stabilization of the myco-synthesized Cu-NPs. The samples were scanned using infrared in the range of 4000:400 cm^−1^ using a FTIR spectrometer (Agilent system Cary 630 FTIR model). The obtained spectral data were compared with the reference chart to identify the functional groups present in the sample (Nandiyanto et al. 2019). The crystalline structure of the myco-synthesized Cu-NPs was characterized by an X-ray diffractometer (XRD-6000 series by Shimadzu apparatus) in order to determine its elemental composition. Scanning electron microscopy (SEM) instrument provides details of sample high-resolution images by scanning the surface to appear the surface morphology. So, it is used for characterization of material structures. Also, energy dispersive X-ray analyzer (EDAX) is used to provide elemental identification and quantitative compositional information.

### Antimicrobial activity of the myco-synthesized CuO-NPs

The antimicrobial activities of the myco-synthesized Cu-NPs were tested against the following pathogens: *Bacillus cereus* ATCC-12228, *Listeria monocytogenes* ATCC-35152, *Enterococcus faecalis* ATCC-29212, *Pseudomonas aeruginosa*, *Salmonella typhi*, *Escherichia coli* ATCC-25922, *Candida albicans* ATCC-10231, *Aspergillus niger* ATCC- 16888, *Aspergillus flavus* ATCC- MYA 4921, and *Fusarium proleferatum* MPVP 328. The antimicrobial activity was tested by well diffusion agar method according to the previous described by Darwesh and Elshahawy (2021). The tested Cu-NP concentration (200 mg/mL) was tested against amoxicillin as antibacterial reference and nystatin as antifungal reference (200 mg/mL). All samples were performed in triplicate and expressed as average values ± SD. Minimum inhibitory concentration (MIC) also was determined (Mourad et al. 2019).

### Immobilization of CuO-NPs on alginate beads

The composite based on nano-copper was prepared to easy handle of the produced CuO-NPs for wastewater treatment. Briefly, a solution of 3 % sodium alginate prepared under continuously steering was mixed with solution of myco-synthesized CuO-NPs under vigorous steering for 1 h. The prepared mixture was added dropwise into calcium chloride solution (5%) and kept under vigorous steering another 1h after finishing alginate/CuO-NP solution. The produced pellets were washed several times by deionized water and freeze-dried.

### Heavy metal removal and dye adsorption by the myco-synthesized CuO-NPs

The capability of the myco-synthesized CuO-NPs to remove three common heavy metals, i.e., led, chromium, and nickel from a synthetic wastewater, was examined. A 500 ppm of the tested heavy metal ions was prepared using their salts (Cr_2_K_2_O_7_, NiCl_2_, and PbNO_3_). CuO-NPs (produced by *Fusarium oxysporum* OSF18) at concentration of 100 mg/10 mL were applied to remove these ions from their solutions; the removal efficiency was calculated (Powara et al. 2019).

For textile dye adsorption, the same concentration of the myco-synthesized CuO-NPs was added to reactive red azo dye solution (300 ppm) and then incubated under shaking conditions (100 rpm) for 24 h. The decolorization efficiency was calculated as previous description by Darwesh et al. (2014).

### Application of immobilized CuO-NPs for textile industry wastewater remediation

The bioremediation efficiency of CuO-NPs immobilized in alginate beads were applied to a real textile industry wastewater treatment sample (collected from textile manufactory at Kafr El-Dawar region, Egypt). A total of 500 mg of alginate beads containing 200 mg of CuO-NPs was added to 10 mL of the collected wastewater sample. After overnight incubation with shaking (100 rpm), total microbial counts, dye, and heavy metal (Pb, Cr, Ni) removals were determined and compared with untreated wastewater. Disinfection, dye decolonization, and heavy metal removal efficiencies were calculated.

## Results and discussion

### Isolation and screening of copper-resistant fungi

The main objective of this study is to bio-remediate the wastewater effluent from textile industry by producing copper nanostructure using active fungal isolates capable to resist and reduce copper ions. The consecutive enrichment culture technique was relied on PDB medium supplied with a high concentration of copper ions to increase the possibility of obtaining copper-resistant fungal isolates from the 12 previously collected samples. After the processes of enrichment in Cu-PDB liquid and culturing on Cu-PDA plates, 35 fungal colonies from different samples, and whenever possible with different morphologies, were selected for isolation and purification. In this hypothesis, the sample source is a key to success of targeted fungi isolation (Vimbela et al. 2017). Screening of the purified fungal isolates either on PDA plates or liquid medium (PDB) supplemented with 1000 ppm CuSO_4_.5H_2_O revealed the superiority of 13 isolates to grow well and tolerate Cu^2+^ (unpublished data). The selected isolates were encoded as F5, F8, F10, F14, F18, F20, F22, F24, F26, F27, F28, F29, F30, and F34.

### Screening for extracellular biosynthesis of copper oxide NPs

**Fig. 1:**
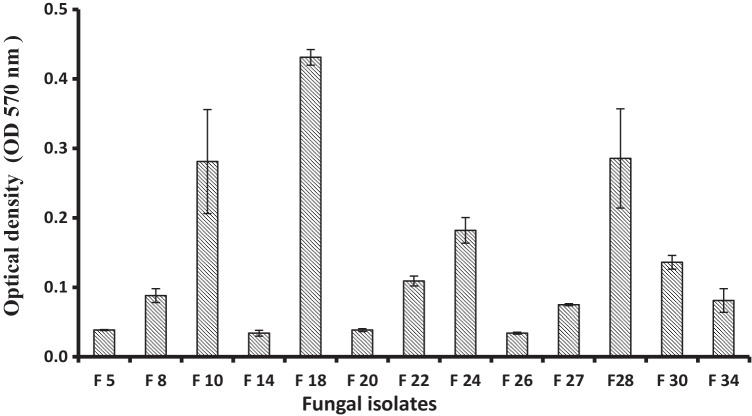
Copper ions reducing and nanostructure formation by fungal isolates at 5000 ppm of copper sulfate

The previously selected 13 Cu-resistant fungal isolates have been screened against the ability for reducing copper ions into its nanoform. Fungi have the ability to produce, both internally and externally, relatively large amounts of proteins and enzymes, which are important in the rapid and sustainable synthesis of nanoparticles (Guilger-Casagrande and Lima 2019). Hence, 3-day-old culture filtrate (on PDB) was tested as a potential medium containing biological reducing agents to convert Cu^2+^ into its nanostructures. The screening was based on the production of precipitated CuO-NPs that can be spectrophotometrically measured at a wavelength of 570 nm (OD_570_) (Fig. S3). The obtained results showed, as in Fig. 1, the variability between the investigated fungal isolates based on the production of CuO-NPs. In solutions containing 5000 ppm CuSO_4_.5H_2_O, the fungal isolate F18 showed greater efficiency in the extracellular biosynthesis of CuO nanoparticles than others. This efficiency can be attributed to the efficient ability to secrete extracellular molecules capable of converting copper ions into their reduced nanoscale form. The reduction system of microbial biomass plays a vital role in reduction of metal ions and fabrication of nanometals’ form (Sungur and Gülmez 2015). It is important to mention that this fungus (F18) was isolated from wastewater of the textile industry, which contained copper (and others) as contaminants, and also showed the highest tolerances to copper ions. This leads to the confirmation that the sample source is of importance in characteristics and a key to success of the targeted microbial isolates (Mani et al. 2021). The most active fungal isolate were obtained from the wastewater samples collected from textile plants. Sungur and Gülmez (2015) reported that the residues of copper and chromium in dyestuffs are possible from the use of catalysts in the synthesis of some dye intermediates. Also, some reactive dyes contain copper, cobalt, nickel, and chromium as metal complexes or metallic impurities, originating from the raw materials used in manufacture.

### Identification of the most active fungal isolate

**Fig. 2:**
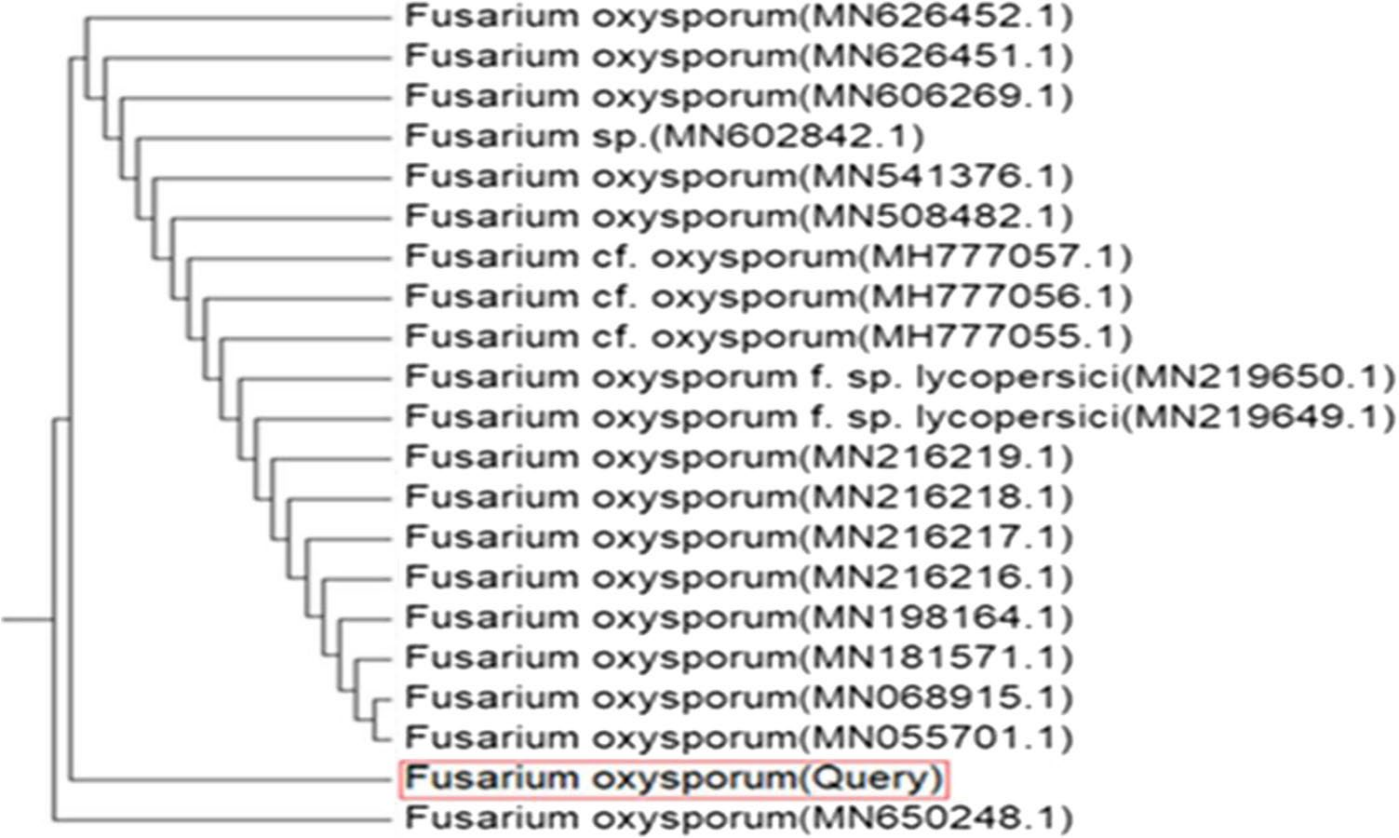
Phylogenetic tree of *F. oxysporum* (OSF18) with the close related reference strains

Based on the tolerance to copper ions and the extracellular biosynthesis of CuO-NPs, the isolate-coded F18 has been selected for identification and further experiments. Cultural and morphological characteristics suggested that this isolated fungal was initially identified as *F. oxysporum* (Nel et al. 2006; Elshahawy et al. 2018). The molecular identification was applied based on the amplification and sequencing of the internal transcribed spacer (ITS) genes. The obtained sequences were compared with sequences available in GenBank using BLAST program (http://www.ncbi.nlm.nih.Gov/BLAST). The phylogenetic relationship showed that this strain is very close to the type strains of *F. oxysporum* (Fig. 2) with a similarity percentage of 98.83 % with other related strains in GenBank*.* The fungus was recorded in GenBank as *F. oxysporum* (OSF18) with accession No. MZ833458.

### Characterization of biosynthesized Cu-NPs

**Fig. 3:**
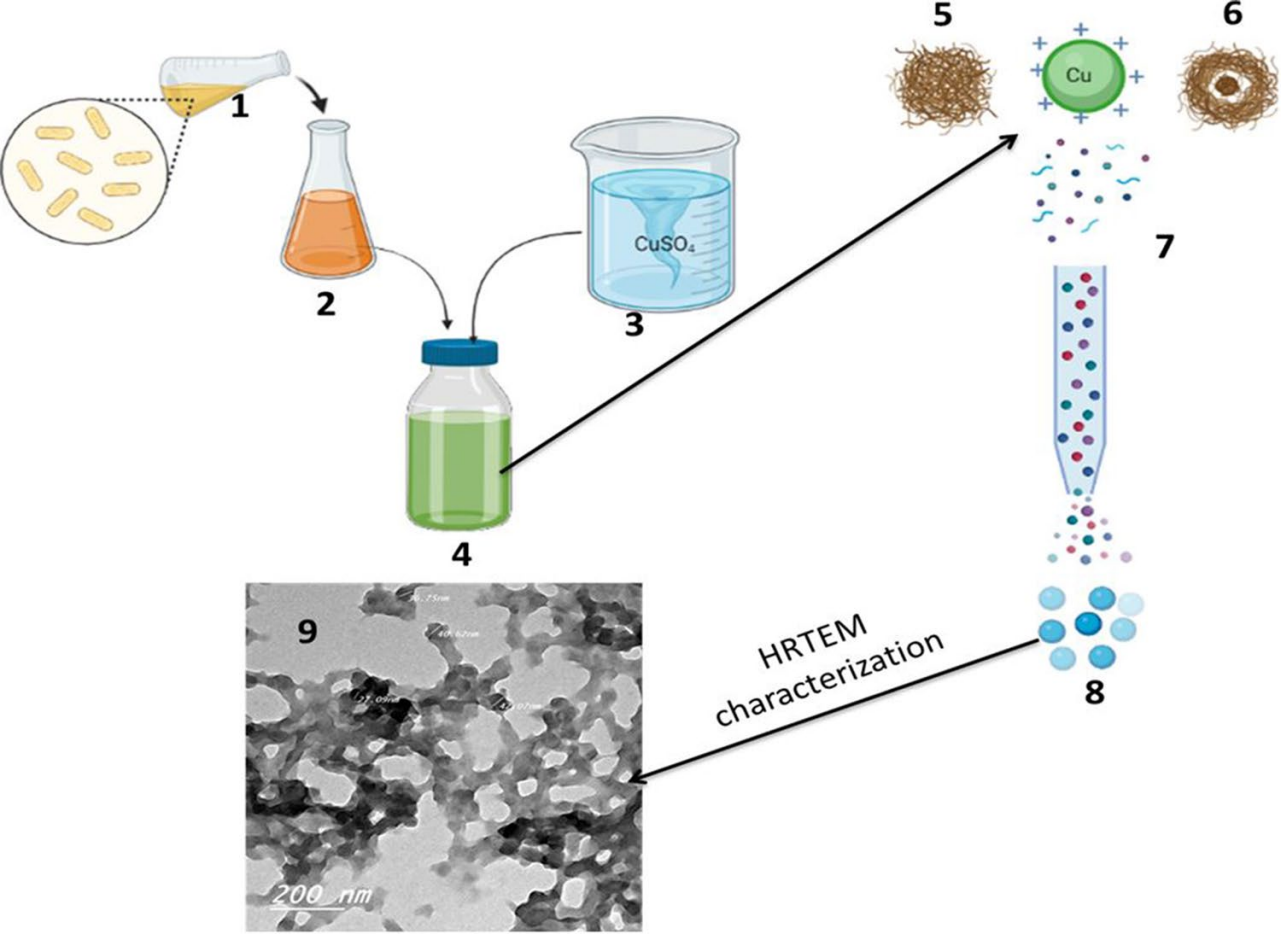
Production and HRTEM characterization of fungal CuO-NPs: 1, fungal culture; 2, culture filtrate; 3, CuSO_4_ solubilization; 4, mixture of Cu ions with filtrate; 5, proteins or reducing agents; 6, reducing agents coating ions; 7, reducing Cu ions; 8, CuO-NPs; 9, HRTEM picture

Copper nanoform produced by *F. oxysporum* (OSF18) was characterized using transmission electron microscopy, Fourier transform infrared spectroscopy, and X-ray diffraction spectrophotometer. Transmission electron microscopy (TEM) is considered the main technique for nano-size material characterization (especially for morphological characterization), because it illustrates the size and morphology of nanoforms (Mourad et al. 2019). In this study, high-resolution transmission electron microscopy (HRTEM) was applied for the morphological analysis of myco-synthesized CuO-NPs. Micrographs obtained from HRTEM (Fig. 3) indicated that the sizes of the CuO-NPs produced ranged from 21 to 47 nm. Regarding the shape, the resulting CuO-NPs had a spherical shape with marginal difference with some aggregates as shown in Fig. 3.

In addition, HRTEM image indicates the presence of a thin layer around the formed CuO-NPs which could be fungal proteins that acts as capping and/or protective agents. These proteins are supposed to be useful for protecting the produced nanoparticles from aggregation. This result agrees with the results reported by previous study (Nabila and Kannabiran 2018; Darwesh et al. 2019d). The observations in this experiment confirm the presence of reducing substances and capping agents in the cell-free extract of *F. oxysporum* (OSF18), indicating the importance of this fungus in the biosynthesis of CuO-NPs.

**Fig. 4:**
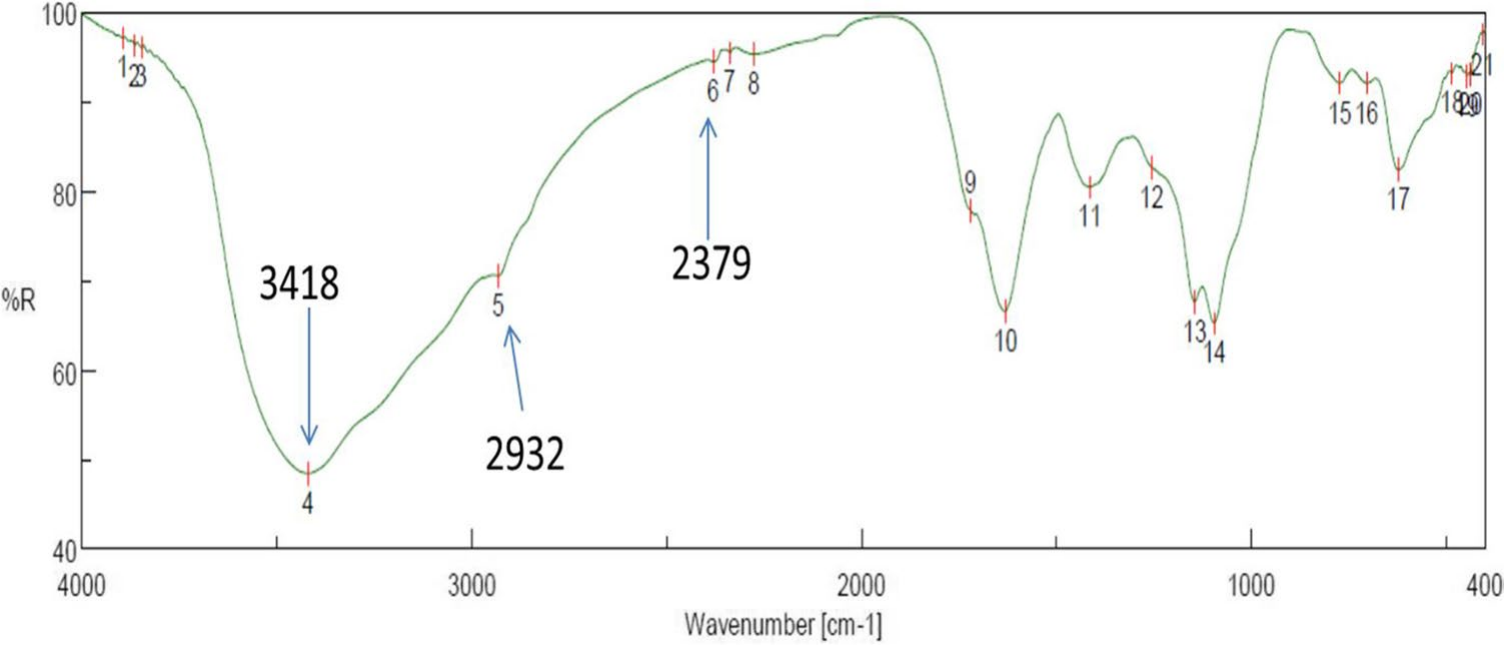
FTIR analyses of the myco-synthesized CuO-NPs

**Fig. 5:**
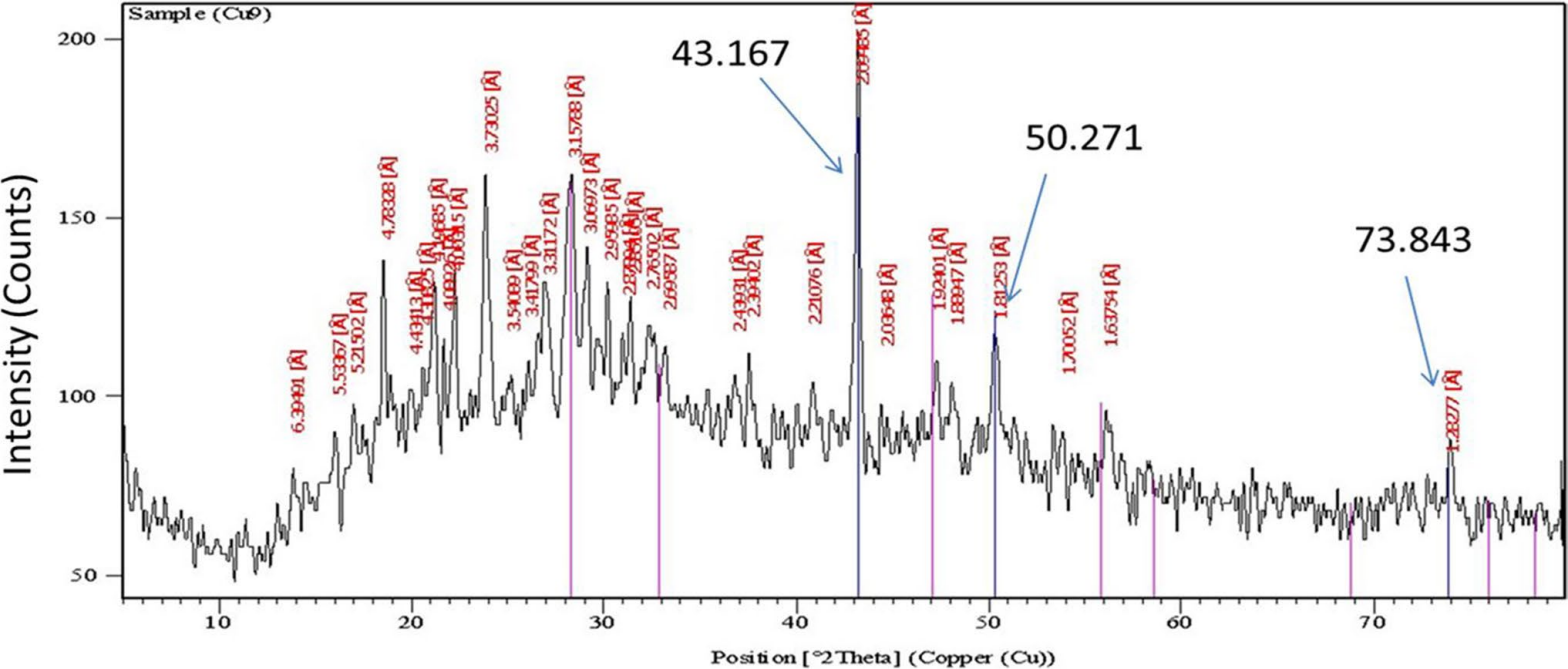
XRD analysis of the myco-synthesized CuO-NPs

FTIR was performed to give insights of functional groups as reducing, stabilizing, and capping agents in the synthesized CuO-NPs. As shown in Fig. 4, the FTIR spectrum in the range of absorption bands from 400 to 4000 cm^−1^ represents the infrared spectrum of CuO-NPs with some vibration bands at different wavelengths. The peak displayed around the range of 600 cm^−1^ refers to the band of alkyl halides (C-Cl) (Rasheed et al. 2017) as in position of 15–18. In contrast, C-H bond peak could be distinguished at 2932 cm^−1^ as a result of methylene, methoxy, and methyl groups stretching vibrations as in position of 5, while O-H group stretching due to the proposed presence of alcohols, flavonoids, and phenols was viewed at 3418 cm^−1^ (Rohaeti et al. 2015), as in position of 4, whereas C=O bond stretching peak was detected at 2379 cm^−1^ (Tungmunnithum et al. 2018) as in position of 6. Based on the peaks of FTIR spectroscopy, protein molecules (possibly active enzymes) are suggested as reducing and capping agents for the biosynthesis of CuO-NPs. The crystallinity nature of the biosynthesized CuO-NPs was identified and confirmed by XRD analysis at peak position with 2θ values (Fig. 5) (Velsankar et al. 2019; Kaliammal et al. 2021).

**Fig. 6:**
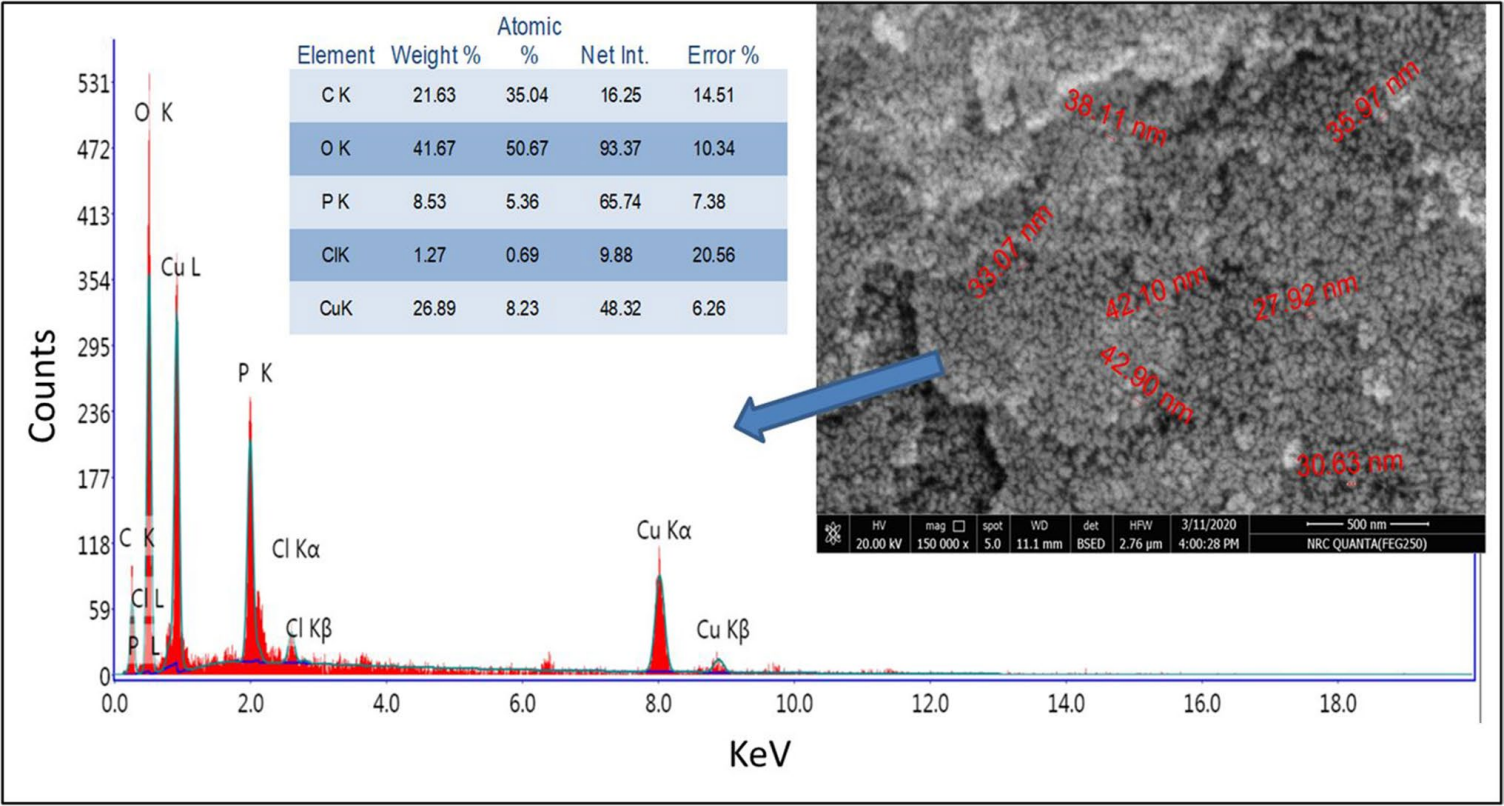
SEM/EDAX characterization of fungal CuO-NPs

The morphology, surface uniformity, and elemental composition of the produced CuO-NPs were characterized using SEM/EDAX and the resulted image and analyses are illustrated in Fig. 6. Results indicated that the surface of CuO-NPs was uniform and belonged to nano size (< 43 nm). This result is in agreed with those obtained by the three-dimensional electron microscopy (HRTEM). For the metal composition of the sample, the EDAX analyses confirmed that the produced is copper oxide nanoparticles coated by protein molecules as appeared in metal analyses (Cu, O, C, P).

### Antimicrobial activity of the biosynthesized CuO-NPs

**Fig. 7:**
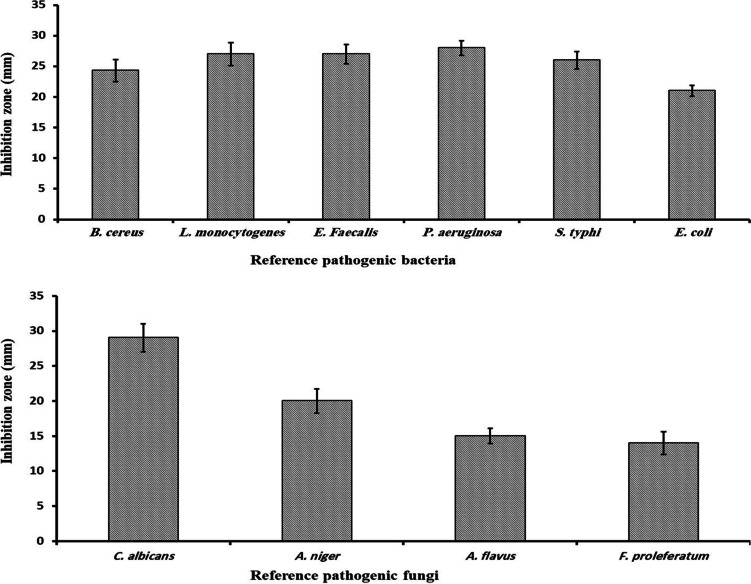
Antimicrobial activity of CuO-NPs (200 mg mL^−1^) produced by *F. oxysporum* (OSF18)

The antimicrobial activity of CuO-NPs biosynthesized using supernatant of *F. oxysporum* (OSF18) was investigated against ten microorganisms using the method of agar well diffusion by measuring the inhibition zones. The tested bacteria were *L. monocytogenes* and *B. cereus* as Gram positive bacteria and *E. coli*, *S. typhi*, and *Ps. aeruginosa* and *E. faecalis* as Gram-negative bacteria. In addition, the yeast *C. albicans*, as well as the fungi *A. niger*, *A. flavus*, and *F. proleferatum*, were tested as representatives of molds. The biosynthesized CuNPs was tested at the concentration of 200 mg/mL and it exhibited noticeable inhibition for the all tested microorganisms as illustrated in Fig. 7. Similar studied on CuO-NPs showed similar results toward pathogenic microorganisms (Longano et al. 2011; Mahmoodi et al. 2018; Sadek et al. 2018; Velsankar et al. 2020c). The antimicrobial activity of CuNPs can be attributed to their large active surface area and thus their ability to interact with pathogens (Qiu et al. 2020; Velsankar et al. 2020b).

The minimum inhibition concentrations (MIC) of CuO-NPs biosynthesized by cultural filtrate of *F. oxysporum* (OSF18) were examined and the obtained values are represented in Table 1. The results showed that the recorded MIC of tested nanoparticles ranged between 20 and 70 mg/mL. These properties and capabilities make CuO-NPs a potential candidate for bioremediation and pathogen control in many medical and environmental fields (such as wastewater treatment).

**Table 1:** Minimal inhibitory concentration (MIC) of the produced CuO-NPs

Pathogenic microorganisms	MIC of CuO-NPs produced by *F. oxysporum* (mg mL^−1^)
***B. cereus***	25
***L. monocytogenes***	25
***E. faecalis***	20
***P. aeruginosa***	33.3
***E. coli***	20
***S. typhi***	25
***C. albicans***	20
***A. niger***	50
***A. flavus***	60
***F. proleferatum***	70

### Heavy metal removal and dye adsorption by myco-synthesized CuO-NPs

**Fig. 8:**
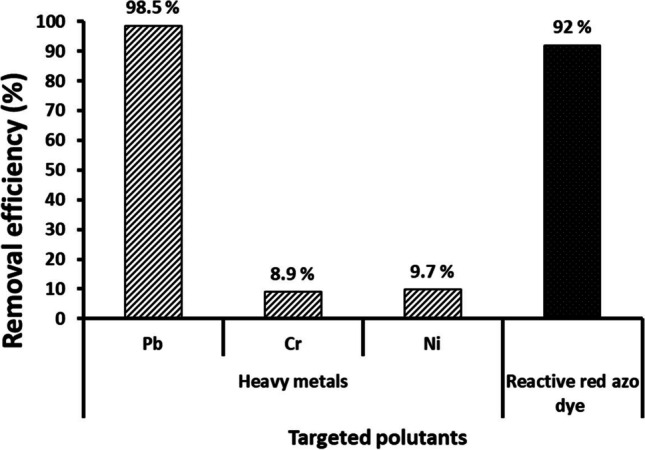
Removal efficiency of Pb, Cr, Ni, and reactive azo red dye from synthetic wastewater by CuO-NPs (10 mg mL^−1^) biosynthesized by *F. oxysporum* (OSF18)

The capability of the biosynthesized CuO-NPs to remove heavy metals and textile dyes from synthetic wastewater was measured. Three solutions of heavy metals, Cr, Pb, and Ni at a concentration of 100 ppm plus a 300 ppm solution of reactive azo red dye, were prepared and used (separately) as the target industrial wastewater for treatment. Bio-synthesized CuO-NPs at concentration of 100 mg/10 mL was applied in the mentioned synthetic wastewater for 24 h and the percentages of heavy metals or dye removal are measured and illustrated in Fig. 8. Regarding the removal of heavy metals by CuO-NPs, although the removal efficiency of chromium and nickel did not exceed 10%, the percentage was much higher in the case of lead (98.49%). Heavy metal removal via nanoparticles mainly occurs due to adsorption onto its surface (El-Dib et al. 2020; Velsankar et al. 2020a; Velsankar et al. 2022b; Darwesh et al. 2021). The adsorption affinity between CuO-NPs and different heavy metals present in the environment varies according to the element, and thus, the removal efficiency of different elements varies under the same conditions.

On the other hand, the color removal of reactive red dye from the synthetic wastewater by the action of CuO-NPs reached 92% within 24 h (Fig. 8). The results indicate the effective ability of the biosynthesized CuO-NP compounds to absorb and decolorize the synthetic dyes, which is one of the main pollutants in textile dye wastewater (Tappe et al. 2000). The wastewater contains textile dyes, heavy metals, and pathogenic microbes as a source of hazardous pollutants, so we focused on removing dyes and heavy metals in addition to control the pathogens.

### Bioremediation of raw textile wastewater using myco-synthesized CuO-NPs

**Fig. 9:**
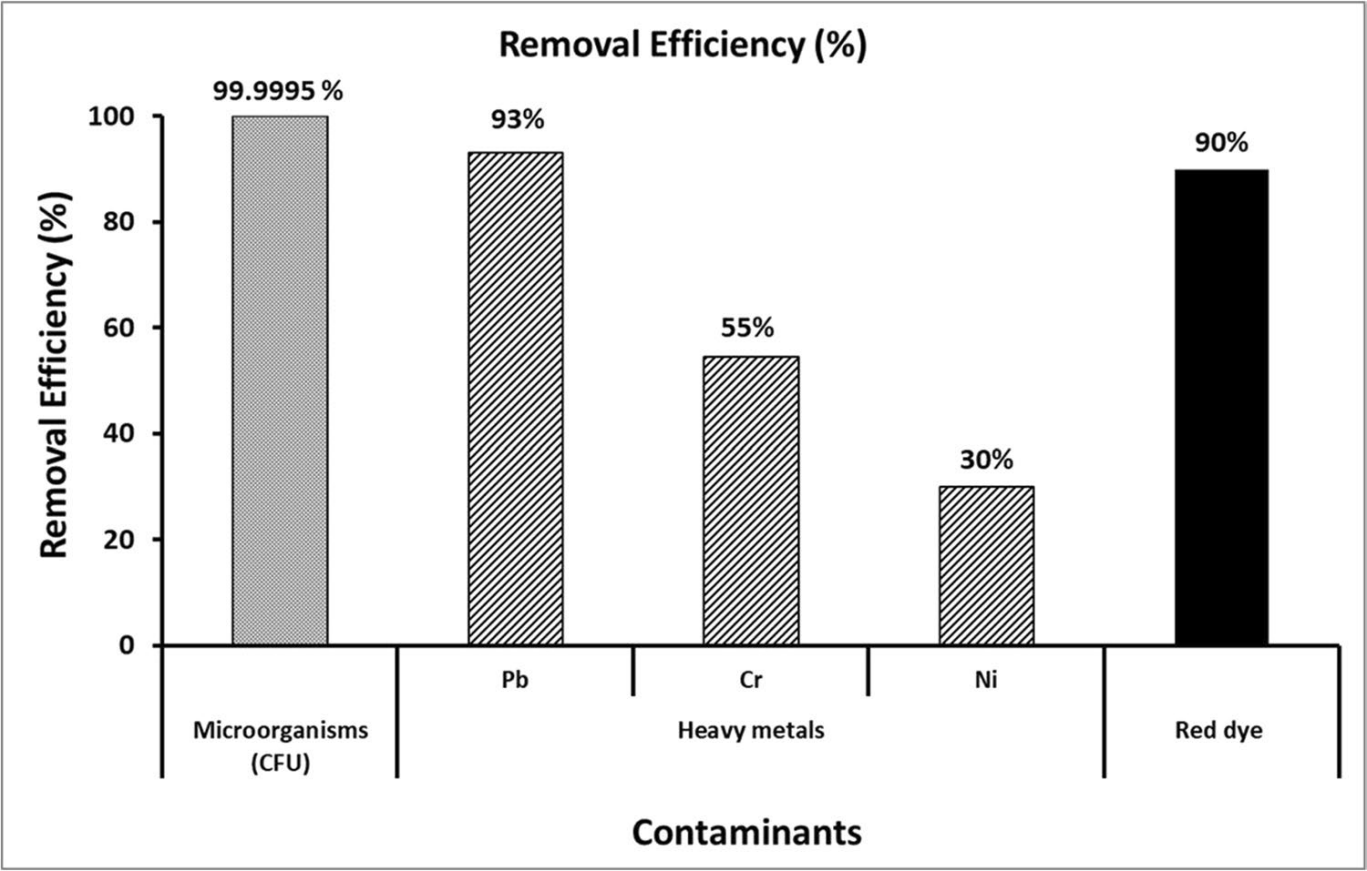
Removal efficiencies of some contaminants from raw textile industry wastewater using biosynthesized CuO-NPs immobilized in alginate beads after 24 h

Previous experiments demonstrated the ability of biosynthetic copper nanoparticles to control pathogenic microbes in addition to absorb and remove heavy metals and dyes from synthetic wastewater. Therefore, it was reasonable to study its application in bioremediation of raw textile wastewater. In industrial applications, it is necessary to immobilize bio/catalyst materials such as enzymes and nanoparticles in suitable support material(s) to protect them against washing-out and adverse surrounding conditions. Hence, in this study, the biosynthesized CuO-NPs were immobilized in alginate beads and applied to treat raw effluent wastewater obtained from a textile factory in Egypt. The level of biological contamination of studied wastewater was measured by microbial counting as colony forming unit (CFU) which was 130 × 10^7^. After treatment, the microbial load was decreased by the percentage of 99.995% (Fig. 9) which can be considered as effective disinfection or partial sterilization of the raw wastewater. In the same raw textile wastewater, the heavy metal removals were as 93, 55, and 30 % for Pb, Cr, and Ni, respectively (Fig. 9). It should be noted that the heavy metal removal in raw textile wastewater took the same pattern as in synthetic wastewater. Also, the removal efficiency for the tested heavy metals was Pb > Cr > Ni which was similar as reported in other studies (Mahmoud et al. 2021; Velsankar et al. 2022a). Regarding dye removal, as in Fig. 9, the percentage was as 90% which almost similar to the percentage obtained from synthetic wastewater.

Textile industry wastewater is produced by huge amounts daily and considered one of the most pollutants producers in world country. Due to increase the pure water shortage, bioremediation of such wastewater is very important to fill the water deficit. From various technologies used in this field, nanotechnology especially from biological source is sustainable developed to treat industrial wastewater and provide non-traditional irrigation water resources (Darwesh et al. 2019a). The obtained results indicated a good activity toward harmful microorganisms. Some other studies reported the antimicrobial activity of CuNPs, and it has been talented for medicine and dentistry fields due to their properties, specially their interaction with pathogens, their large active surface area, and their high chemical and biological reactivity (Longano et al. 2011; Mahmoodi et al. 2018; Darwesh et al. 2019e). When their surface is appropriately elaborated, CuNPs could also provide efficient binding to the bacteria and/or other pollutants (heavy metals or dyes) because their high surface/volume ratio simply offers more contact area. Finally, the comparison between obtained CuO-NPs in the current study and other literature biosynthesized CuO-NPs are illustrated in Table 2.

**Table 2:** Comparison between obtained CuO-NPs in the current study and other literature biosynthesized CuO-NPs

		Biological synthesizing agent	Particle size	Suggested capping agent	Application(s)	Ref.
Our bio-synthesized CuNPs		*Fusarium oxysporum* OSF18	Spherical nanocrystals/21–47 nm	protein molecules (possibly active enzymes) are suggested as reducing and capping agents	Removing heavy metals (93, 55, and 30 % for Pb, Cr, and Ni, respectively) and textile dye (90%) from industrial wastewater Disinfection of wastewater (99.995% efficiency)	Our data
Literature’s bio-synthesized CuNPs	Bacteria	bacterial consortium: *Marinomonas*, *Rhodococcus*, *Pseudomonas*, *Brevundimonas*, and *Bacillus* (BC)	Spherical CuONP/of 30 nm	proteins	antimicrobial activity against various types of Gram-negative; Gram-positive bacteria; and fungi pathogen microorganisms including *Escherichia coli*, *Staphylococcus aureus*, and *Candida albicans*.	(John et al. 2021)
	Fungi	*Stereum hirsutum*	CuO-NPs spherical (5 to 20 nm)	Polycarbohydrate and protein	Not mentioned	(Cuevas et al. 2015)
		*Aspergillus niger*	500 nm	Functional alkenes groups	Biomedical applications: cytotoxic effect against human hepatocellular carcinoma cell lines (IC50 value was determined as 3.01 μg/ml); antibacterial effect	(Noor et al. 2020)
		*Hypocrea lixii*	Spherical/24.5 nm	Proteins (amide groups)	Suggested for bioremediation of wastewater	(Salvadori etal. 2013)
		*Cantharellus* sp.	Spherical/72.4 nm.	Phenolic group	Antimicrobial activity	(Jha et al. 2021)
		*Penicillium olsonii*	6–26 nm	Polyphenolic O-H group and primary amine O-H band.	Antifungal activity (tested against *F. oxysporum*, *F. solani*, and *C. curvulatat* with growth inhibition 86.25, 32.92, and 68.42%, respectively, at 200ppm)	(Abboud 2021)
	Actinomycetes	*Streptomyces capillispiralis*	Spherical-monodispersed-shaped CuNPs/3.6–59 nm	Proteins and carboxylic residues	Biomedical applications against infectious microorganisms, biocontrol of phytopathogenic fungi, and health nasty insects	(Hassan et al. 2018)
	Microalgae	*Botryococcus braunii*	Cubical and spherical with an elongated shape CuO-NPs/10–70 nm	Protein	Antibacterial activity (against both bacteria (Gram positive and Gram negative) and fungus)	(Arya et al. 2018)
	Plant	*Terminalia arjuna*	23 nm	Plant metabolites such as flavonoids, proteins, terpenoids, tannins, and polyphenols	Antibacterial activity	(Yallappa etal. 2013)

## Conclusions

Bionanotechnologies received great interest due to their hopeful results and potential benefits on many life aspects. In this study, we report the eco-friendly and cost-effective biosynthesis of CuO-NPs using the cell-free extract of a novel copper-resistant fungus strain, *F. oxysporum* OSF18. The biosynthesized CuO-NPs were characterized and found to be spherical nanocrystals with the size range of 21–47 nm. Bio-CuO-NPs immobilized in alginate beads exhibited a great efficiency in disinfecting microbes (99.995%) and removing heavy metals (93, 55, and 30 % for Pb, Cr, and Ni, respectively) and dyes (90%) from raw textile industrial wastewater. The biosynthesized CuO-NPs immobilized in alginate beads could be concluded as eco-friendly, cost-effective, and easy to handle for the bioremediation of textile industry wastewater. More research is needed to study the potential long-term impact on the environment when such materials are used on an industrial scale.

## Supplementary Information

ESM 1 (DOCX 11090 kb)

## Data Availability

It is available when requested.
