# Supplementary material for: Nano-bioremediation of textile industry wastewater using immobilized CuO-NPs myco-synthesized by a novel Cu-resistant Fusarium oxysporum OSF18
Source: Environ Sci Pollut Res Int. 2022 Oct 3;30(6):16694–706. doi: 10.1007/s11356-022-23360-7 (PMC9908718; doi:10.1007/s11356-022-23360-7)
Supplement: Supplementary file 1 — (DOCX 11090 kb) [file 11356_2022_23360_MOESM1_ESM.docx]

**Supplementary data**

Fig. S1. FTIR analyses of the chemical synthesized CuO-NPs





Fig. S2. TEM image of the chemical synthesized CuO-NPs


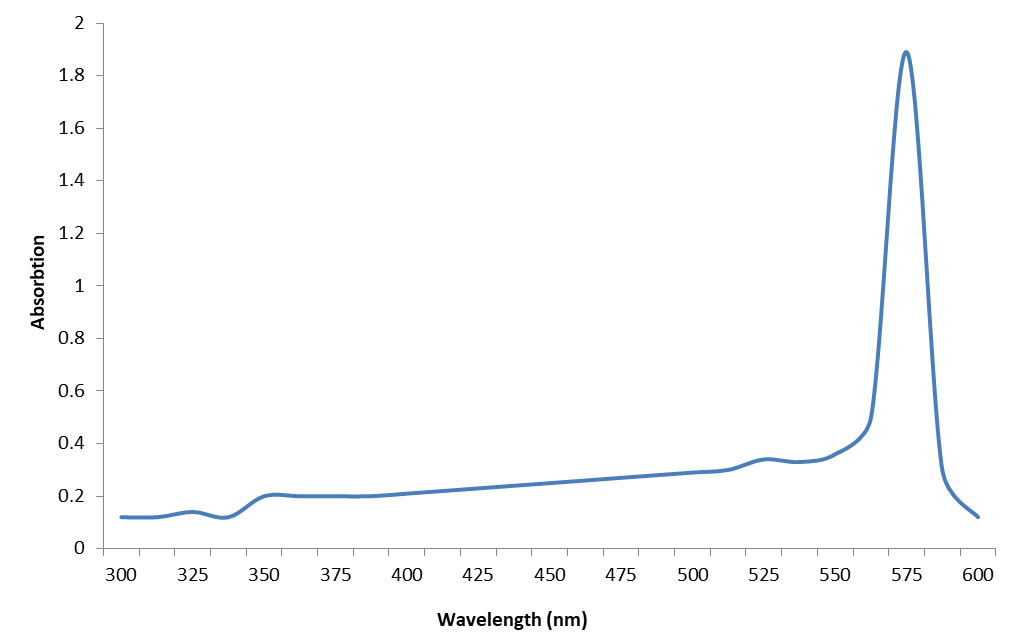


Fig. S3. UV-vis spectrum of bio-synthesized CuO-NPs


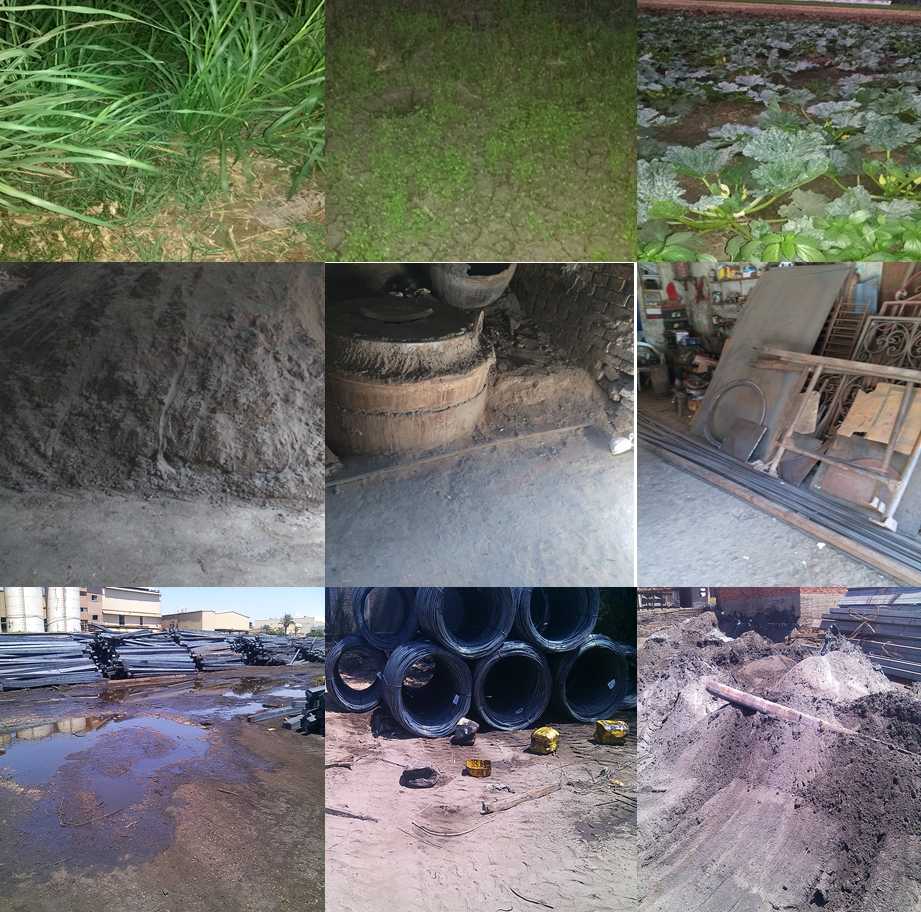


Fig. S4. Source of samples used for fungi isolation.


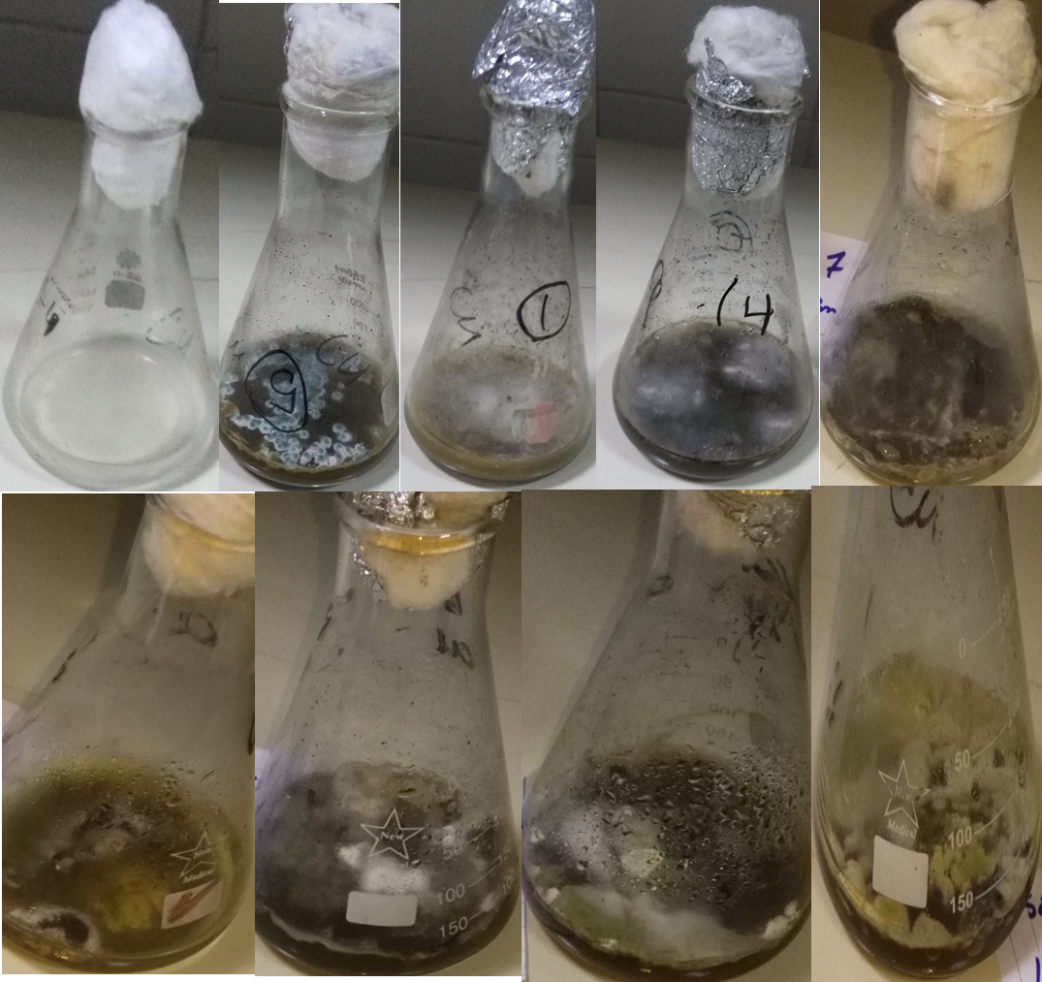


Fig. S5. Some enrichment cultures containing fungi communities.


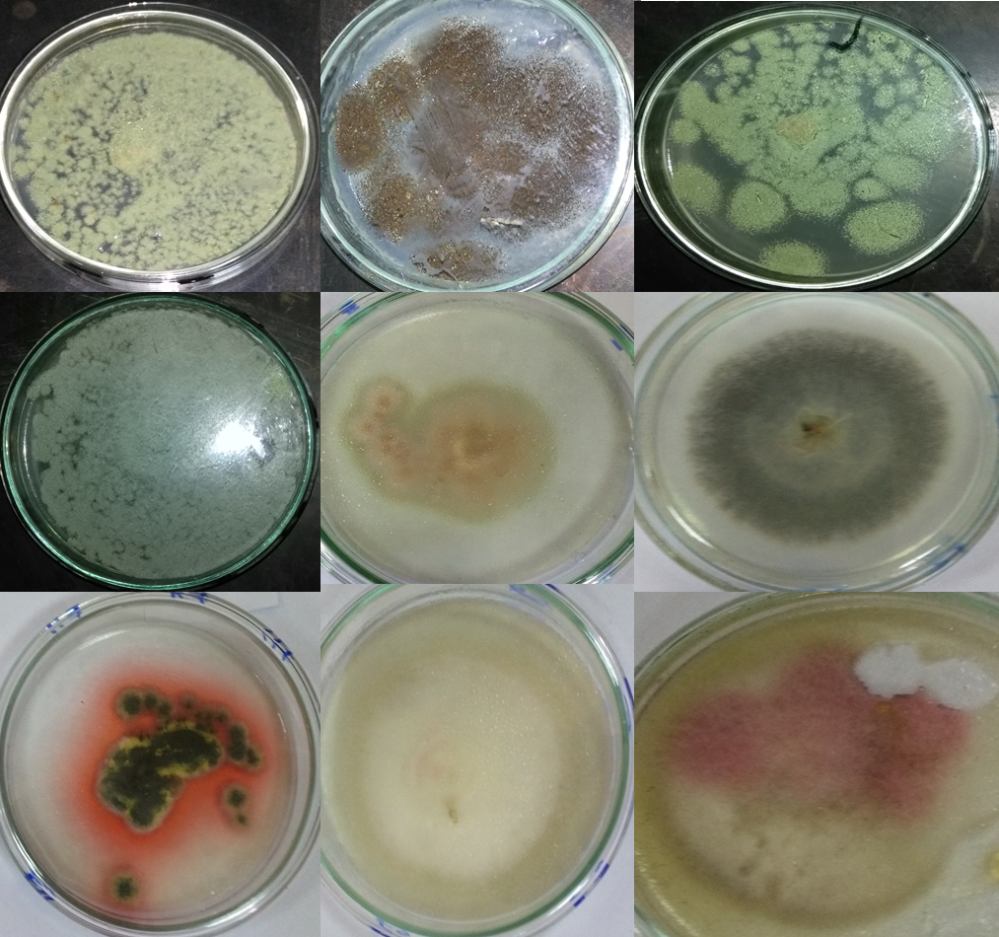


Fig. S6 The morphology of some fungal isolates onto agar plates.

**
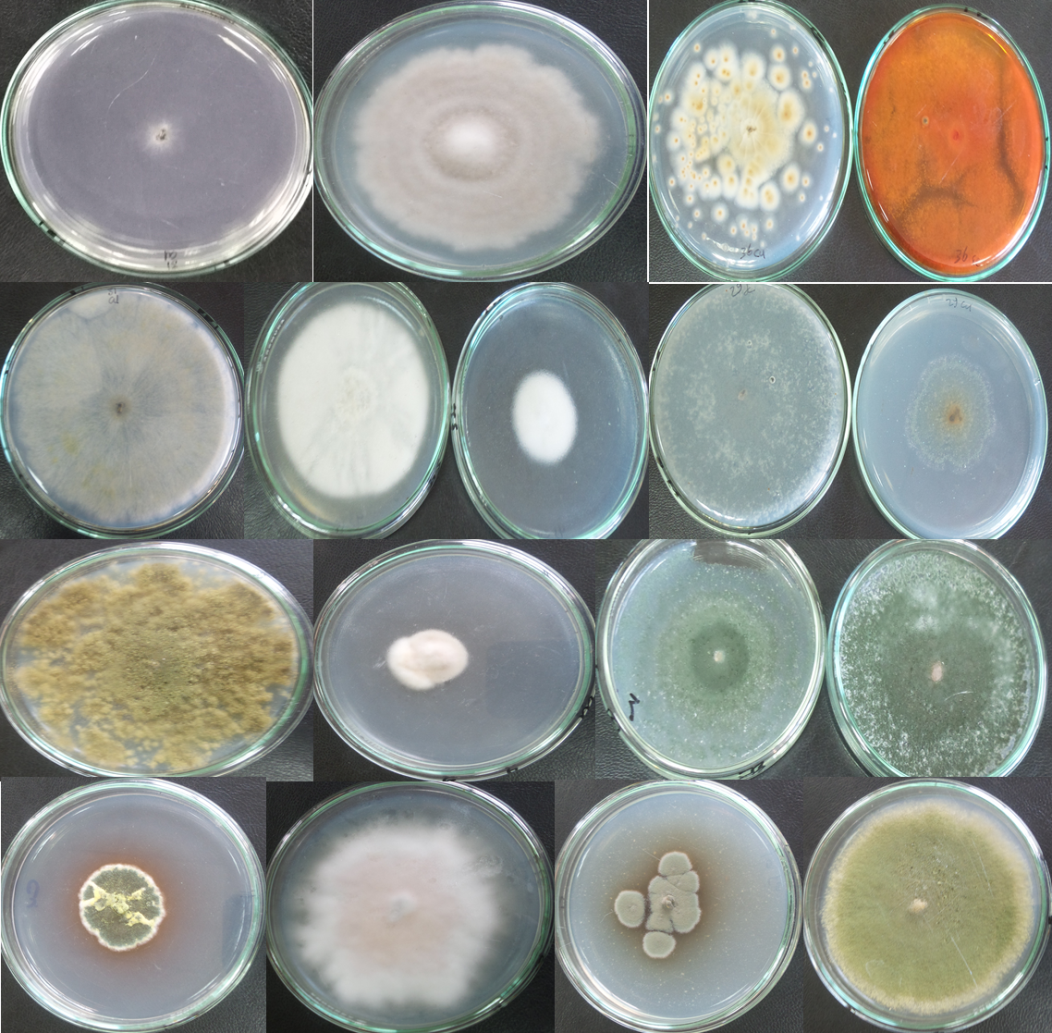
**

Fig. S7 Ability of fungal to grow onto media contained 0.1 % of CuSO_4_ or FeCl_3_ determined as growth diameter
